# Optimal Sound Presentation Level for Sound Localization Testing in Unilateral Conductive Hearing Loss

**DOI:** 10.3390/audiolres15040095

**Published:** 2025-08-02

**Authors:** Miki Takahara, Takanori Nishiyama, Yu Fumiiri, Tsubasa Kitama, Makoto Hosoya, Marie N. Shimanuki, Masafumi Ueno, Takeshi Wakabayashi, Hiroyuki Ozawa, Naoki Oishi

**Affiliations:** 1 Department of Otorhinolaryngology-Head and Neck Surgery, Keio University School of Medicine, 35 Shinanomachi, Shinjuku-ku, Tokyo 160-8582, Japan; miki.t.k@keio.jp (M.T.);; 2 Otology and Audiology Center, Keio University Hospital, 35 Shinanomachi, Shinjuku-ku, Tokyo 160-8582, Japan

**Keywords:** sound localization test, unilateral conductive hearing loss, interaural attenuation

## Abstract

**Background/Objectives**: This study aimed to investigate the optimal sound presentation level for sound localization testing to assess the effect of hearing interventions in individuals with unilateral conductive hearing loss (UCHL). **Methods**: Nine participants with normal hearing were tested, and simulated two-stage UCHL was created using earmuffs and earplugs. We created two types of masking conditions: (1) only an earplug inserted, and (2) an earplug inserted with an earmuff worn. A sound localization test was performed for each condition. The sound presentation levels were 40, 45, 50, 55, 60, 65, and 70 dB SPL, and the results were evaluated using root mean square and d-values. **Results**: Both values showed little difference in masking Condition 2, regardless of the sound presentation level, whereas in masking Condition 1, the values were at their minimum at 55 dB SPL. In addition, comparing the differences between masking Conditions 1 and 2 for each sound presentation level, the greatest difference was observed at 55 dB SPL for both values. **Conclusions**: The optimal sound presentation level for sound localization testing to assess hearing intervention effects in UCHL was 55 dB. This result may be attributed to the effect of input from the non-masked ear, accounting for interaural attenuation; the effect was considered minimal at 55 dB SPL.

## 1. Introduction

Patients with unilateral hearing loss have poor sound localization abilities [1]. A previous report showed that hearing aids can improve sound localization ability in patients with unilateral hearing loss through the benefits of binaural hearing [2].

One of the major diseases of conductive hearing loss is aural atresia. Aural atresia frequently occurs with microtia, and 77–93% of microtia cases are unilateral [3]. Typically, it results in conductive hearing loss of around 50–60 dB in the impaired ear [4]. Because of the deficit in the external auditory meatus, conventional air conduction hearing aids are either difficult to wear or ineffective [5]. To utilize the auditory function of the impaired side, alternative hearing devices such as bone conduction hearing aids, bone conduction implants (e.g., BAHA, BONEBRIDGE), and middle ear implants (e.g., VSB) are often employed.

The cartilage conduction hearing aid (CCHA), released in Japan in 2017, is an appropriate device for patients with aural atresia in whom conventional air or bone conduction hearing aids are ineffective [6]. Cartilage conduction has been reported as a third auditory pathway, different from air and bone conduction [7]. Stimulating the cochlea through the external cartilage, CCHAs theoretically enable bilateral hearing. Indeed, previous reports have demonstrated benefits such as binaural summation [8] and improved speech perception in noisy environments [9]. CCHAs have been widely used in many patients [10], not only including adults [11], but also children [12], providing a non-surgical option or minimal risk of the skin complications often seen with bone conduction implants while achieving favorable hearing abilities. Recently, a new device has emerged: an auricular prosthesis incorporating a cartilage conduction hearing aid (APiCHA) [13]. APiCHA integrates a highly refined auricular prosthesis created by 3D printing technology with a CCHA. This innovative device is expected to evolve further as it improves both esthetics and auditory function without surgery. Although CCHA is currently only used in Japan, clinical trials of CCHA are being conducted in countries such as the United States [14] and Indonesia [15], and its global adoption is expected in the future.

In this way, as the treatment options for unilateral conductive hearing loss (UCHL) have increased with advancements in technology, the importance of accurately evaluating sound localization ability, which reflects the binaural hearing benefits of these treatments, is also growing.

Sound localization tests are useful for evaluating sound localization abilities. Currently, various methods, including headphone-based and multispeaker tests, are employed to assess sound localization ability. Although multispeaker tests are often employed to evaluate the effects of hearing devices, no globally standardized sound localization testing method has been established.

**Figure 1:**
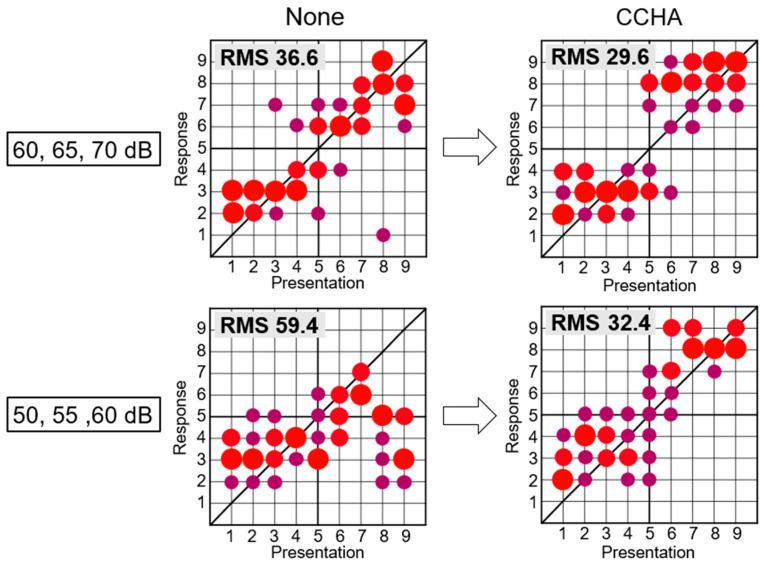
Difference in sound localization test results by the presentation level. The scatter plots show the relationship between the actual sound source and the speaker answered by the participant. The size and darkness of each circle indicate the number of responses at that location. Localization accuracy was evaluated using root mean square (RMS) values, and smaller values indicate better localization accuracy The upper figures show localization test results at higher presentation levels (60, 65, 70 dB SPL), and the lower figures show results at lower levels (50, 55, 60 dB SPL). The differences in RMS values between unaided and aided conditions are greater in the lower figures than in the upper figures.

We previously conducted sound localization tests at 60, 65, and 70 dB SPL using a multispeaker device in patients with UCHL caused by congenital aural atresia both with and without CCHAs. While some patients showed an improvement in sound localization ability with CCHAs, others exhibited little change or even deterioration. In some cases, the patient exhibited good localization ability even without wearing the CCHA, resulting in a small difference from the aided condition, and the benefit of the CCHA was insufficiently demonstrated. When the sound presentation level was lowered to 50, 55, and 60 dB SPL, the same patients exhibited poorer localization ability without CCHAs and significant improvement with CCHAs (Figure 1). This finding suggests that the sound presentation level is an important factor when conducting sound localization tests in patients with unilateral hearing loss.

A previous study similarly reported that listeners with acquired UCHL demonstrated good localization ability without a hearing device when the sound was presented at a high level (65 dB SPL), presumably due to the use of remaining binaural cues. Furthermore, good localization ability was also observed at a low level (45 dB SPL), which is thought to be supported by monaural cues [16]. Thus, even patients with unilateral hearing loss may localize sound sources using binaural difference cues or monaural cues [17].

Monaural cues may be utilized particularly when binaural cues are inaccessible, such as in cases of moderate to severe unilateral hearing loss. These cues include spectral, level, and timbre cues. Spectral cues are related to the shape of the pinna and can be used for horizontal localization even when one ear is occluded [17,18,19]. Additionally, localization may be supported by the overall sound level (level cues) or by the change in timbre resulting from frequency-dependent attenuation due to low-pass filtering by the head (timbre cues). These monaural cues are particularly influential when the presentation level is not roved or is roved insufficiently (i.e., less than 20 dB) [18]. Therefore, if the presentation level is not roved over at least a 20 dB range, monaural cues may be used for localization, which can confound the true assessment of localization ability.

As mentioned above, the ability to utilize monaural cues for sound localization varies among individuals, and sound localization ability can be modified through training [20]. These findings suggest that sound localization is composed of multiple factors. In the present study, we focused on changes in localization performance before and after a hearing intervention. Our aim was to identify the optimal sound presentation level for evaluating changes in sound localization ability in individuals with UCHL by simulating different hearing thresholds to assess the effect of hearing interventions efficiently.

## 2. Materials and Methods

### 2.1. Participants

Nine healthy adults with an average age of 28.8 years (range: 23–37 years; female = 5; male = 4) were enrolled in this study. All participants had pure-tone hearing thresholds < 25 dB HL at seven frequencies (125, 250, 500, 1000, 2000, 4000, 8000 Hz), with no significant bilateral differences. The four frequencies’ (500, 1000, 2000, 4000 Hz) average ± SD (standard deviation) hearing thresholds were 7.1 ± 5.9 dB HL for the left ear and 6.4 ± 6.0 dB HL for the right ear.

This study was approved by the Institutional Review Board of Keio University School of Medicine (approval number: 20140242).

**Figure 2:**
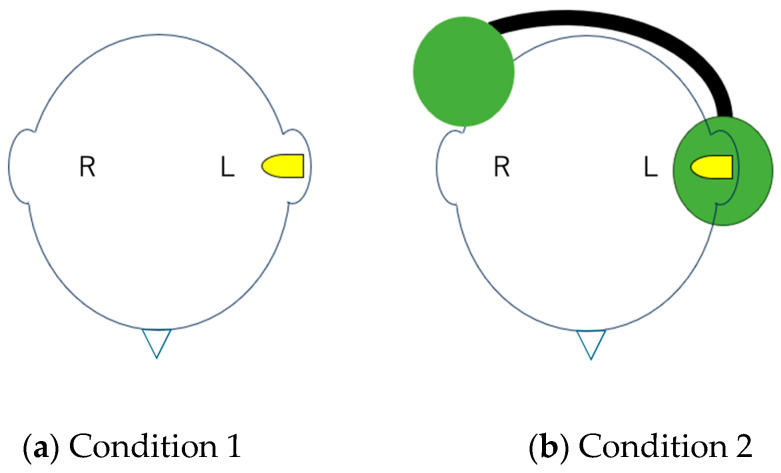
Schema of two types of simulated UCHL. In Condition 1, the left ear was occluded with an earplug only. In Condition 2, the left ear was occluded with both an earplug and an earmuff.

We created two types of UCHL using the following methods: (1) only an earplug was inserted into the left ear (Condition 1) and (2) an earplug was inserted into the left ear and an earmuff was additionally put on the participant (Condition 2) (Figure 2).

### 2.2. Audiological Testing

We conducted sound field audiometry and sound localization tests under both conditions. All audiological tests were conducted in a soundproofed room.

Sound field audiometry was conducted according to the Guidelines for Hearing Aid Fit Testing by the Japan Audiological Society (2010) [21] using a commercially available audiometer (Model AA-H1; RION Co., Ltd., Tokyo, Japan). A loudspeaker was positioned at the same height as the center of the participant’s head, with a distance of 1 m between them. The participant and loudspeaker were placed 1 m away from a wall. The non-test ear (right ear) was masked by narrowband noise through headphones.

A sound localization test was conducted as previously described [22]. Participants were seated in front of nine loudspeakers (6301B, FOSTEX, Tokyo, Japan) arranged at equal intervals of 22.5° in a semicircle of a 1 m radius. Each loudspeaker was numbered sequentially from 1 (left end) to 9 (right end). The stimulus was a 1 s speech-shaped noise (CCITT; comite consultative international telegraphique et telephomique noise) that was randomly presented at varied sound levels via the audio interface (828mk3 Hybrid, MOTU, Cambridge, MA, USA). The presentation levels used in a single test consisted of three levels: the center sound presentation level and levels ± 5 dB SPL from the center. Each level was presented twice from each of the nine speakers, resulting in 54 presentations per test. The center levels were set at 45, 55, and 65 dB SPL, and each participants underwent the test three times for each center level under two masking conditions. Therefore, the presentation levels examined in this study were set at 40, 45, 50, 55, 60, 65, and 70 dB SPL.

Participants were instructed not to move their heads or gaze at the front speaker during the sound presentation. After the sound presentation, the participants answered the number of speakers they thought were presenting the stimulus.

### 2.3. Evaluation Procedures

Sound localization accuracy under each masking condition was evaluated for each presentation level. The outcome measures were the root mean square (RMS) value and the mean deviation score (d-value). The RMS value is the square root of the mean of the squared deviations between the actual sound source direction and the direction indicated by the participant. The d-value is the mean of the absolute values of the deviations. For both measures, smaller values indicate better localization accuracy. The RMS value was evaluated at 10 dB intervals of the center sound presentation levels, whereas the d-value was evaluated at 5 dB intervals of the sound presentation levels to detect subtle changes in localization accuracy. The d-value at a presentation level of 50 dB was included in both tests where the center presentation level was set at 45 dB and 55 dB, and the same applied to 60 dB. Therefore, the values from the two tests were averaged to obtain the d-value at each presentation level.

## 3. Results

### 3.1. Sound Field Audiometry

Table 1 shows the mean ± SD sound field hearing thresholds of all frequencies. The four frequencies’ (500, 1000, 2000, 4000 Hz) average hearing thresholds for the left ear were 43.2 ± 11.6 dB HL under Condition 1 and 61.3 ± 6.5 dB HL under Condition 2.

### 3.2. Sound Localization Test

Due to computer trouble, some missing values occurred. Specifically, one data point was missing for the RMS value at a center presentation level of 45 dB under Condition 1; the d-values at presentation levels 40, 45, 65, and 70 dB under Condition 1; and the d-value at 55 dB under Condition 2. These data were analyzed with the missing values excluded.

Table 2 and Table 3 show the mean RMS values and d-values at each presentation level under two conditions. To compare differences between the conditions, the mean differences in both values at each presentation level are also presented.

**Figure 3:**
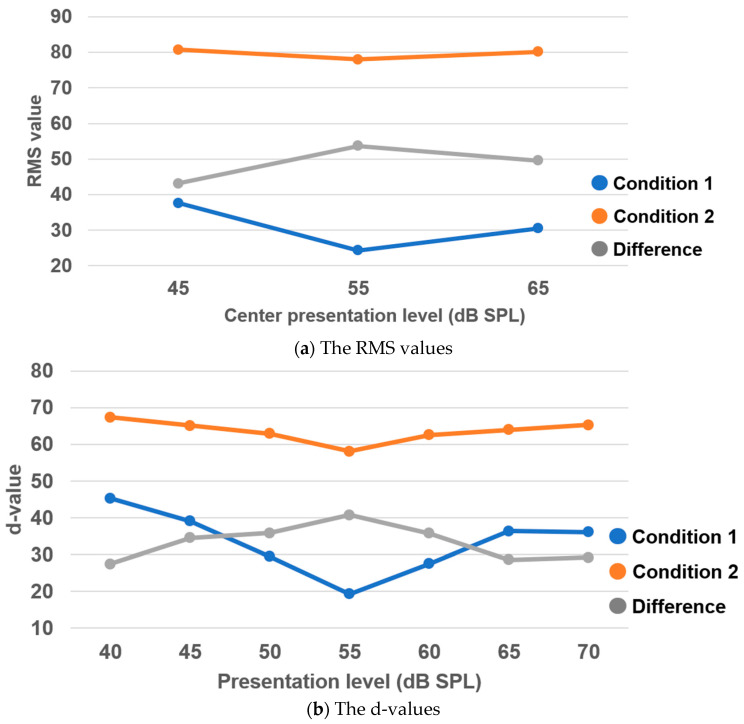
The mean RMS and d-values for each sound presentation level and the differences between the two conditions.

Figure 3 visualizes the results described above. It shows that there were no substantial differences across the presentation levels for either the RMS or d-values in Condition 2; however, in Condition 1, both values were the lowest at 55 dB SPL. Furthermore, when comparing the differences between Conditions 1 and 2 across each presentation level, the greatest difference was observed at 55 dB SPL.

## 4. Discussion

In the present study, we investigated the optimal sound presentation level for sound localization testing in patients with UCHL to accurately evaluate the effects of hearing interventions. Condition 2, in which the ear was occluded with both an earplug and an earmuff, simulated the unaided condition of patients with UCHL, whereas Condition 1, with only an earplug, simulated the aided condition. The results demonstrated that Condition 1 exhibited the lowest RMS and d-values, and the difference between Conditions 1 and 2 was the largest at 55 dB SPL.

The sound presented by the speaker reached both ears. In the left ear (hearing-impaired ear), the directly delivered sound was calculated as the presentation level minus the air conduction (AC) threshold. Because the sound localization test evaluates binaural hearing, the sound received in the right ear (normal-hearing ear) can be transmitted to the left ear via bone conduction, resulting in shadow hearing. The level of sound transmitted from the normal ear to the impaired ear can be estimated by subtracting the interaural attenuation (IA) from the presentation level. In general, the IA for AC testing is reported to be 40–60 dB [23]. Therefore, in this study, we assumed an IA of 50 dB to estimate the input to each ear.

**Figure 4:**
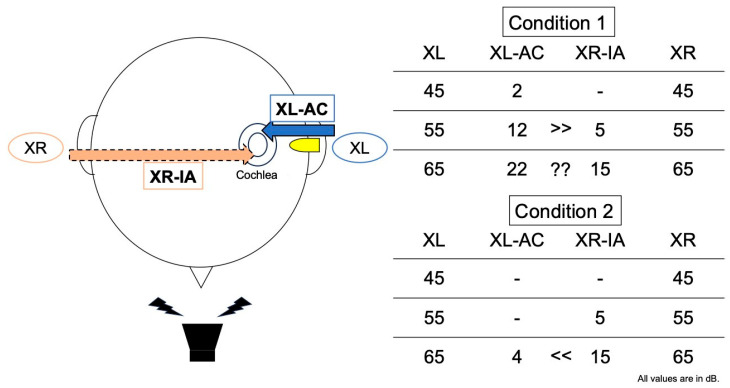
Schema of how IA, AC, and the presentation level interact with the impaired ear and the estimated input levels for each presentation level. X: presentation level, AC: air conduction threshold, IA: interaural attenuation.

At a presentation level of 45 dB SPL, the presentation level was below the AC and IA thresholds in both conditions. Thus, the impaired ear received very little sound, and its localization ability depended largely on that of the normal ear. At a presentation level of 55 dB SPL, the presentation level exceeded both the AC threshold and IA in Condition 1. Therefore, the direct input to the impaired ear was 12 dB, while shadow hearing from the normal ear was 5 dB; therefore, the input from the impaired ear was dominant. In contrast, under Condition 2, the presentation level exceeded the IA but remained below the AC threshold. Therefore, the impaired ear received very little sound directly, and its localization ability depended largely on that of the normal ear. At a presentation level of 65 dB SPL, the level exceeded both the AC threshold and IA under both conditions. In Condition 2, the impaired ear received a slight direct input (4 dB), while shadow hearing from the normal ear was 15 dB; therefore, localization ability still depended on the normal ear. In Condition 1, the direct input to the impaired ear increased (22 dB), and shadow hearing also increased (15 dB), which may have caused confusion in terms of sound localization because there was a certain amount of input from both ears (Figure 4).

Thus, although little difference was observed across presentation levels in Condition 2, at 55 dB SPL, Condition 1 showed the best localization ability and there was largest difference between Conditions 1 and 2. This suggests that 55 dB SPL may be the most balanced presentation level between the direct input to the impaired ear and shadow hearing from the normal ear, and may represent the optimal presentation level for evaluating the effect of hearing interventions.

Although sound localization testing is commonly used to evaluate bilateral hearing after hearing interventions, no standardized method has been established. In previous studies, presentation levels ranged between 60 and 80 dB SPL [24,25,26,27,28], but varied across studies. To the best of our knowledge, no previous studies have clearly identified the optimal presentation level for sound localization tests. Furthermore, as mentioned above, patients with unilateral hearing loss may localize sound sources using various monaural cues. To evaluate true localization ability, it is necessary to rove the presentation level over a range of at least 20 dB [18] to minimize the influence of monaural cues.

In the present study, the roving range was relatively narrow, which may have been insufficient to adequately minimize the influence of particularly level and timbre cues. However, rather than aiming to assess absolute localization ability, this study focused on changes in localization ability before and after hearing intervention, thus demonstrating practical improvements of the localization accuracy. Our findings suggest that 55 dB SPL may be the optimal sound presentation level for evaluating the effects of hearing intervention in sound localization testing for patients with UCHL, providing a new finding in this field.

## 5. Limitations

In the present study, the number of participants was small; therefore the results should be interpreted with caution. Additionally, this study used a simulated UCHL model, and the hearing thresholds may differ from those of actual patients, leading to different results. However, the sound presentation level must represent a realistic sound pressure level that patients are likely to encounter in daily life and should not exceed the IA excessively. From this perspective, we considered 55 dB SPL to be a reasonable presentation level. Future studies involving more participants with various hearing thresholds are required to determine more appropriate presentation levels.

## 6. Conclusions

The present study suggests that a presentation level of 55 dB SPL is optimal for evaluating the effects of hearing interventions on sound localization tests in patients with UCHL. This sound level is considered to be less affected by interaural attenuation and shadow hearing, and provides a balanced input from both impaired and normal-hearing ears.

## Figures and Tables

**Table 1:** The mean sound field hearing thresholds (dB HL) (Mean ± SD).

	125 Hz	250 Hz	500 Hz	1000 Hz	2000 Hz	4000 Hz	8000 Hz
**Condition 1**	36.1 ± 13.6	40.6 ± 12.4	40.0 ± 12.5	40.0 ± 15.4	43.3 ± 12.5	49.4 ± 12.6	53.9 ± 11.1
**Condition 2**	46.1 ± 8.2	51.1 ± 8.6	57.2 ± 7.6	60.6 ± 9.7	58.9 ± 8.6	68.9 ± 4.9	47.2 ± 6.7

**Table 2:** The mean RMS values at each center level (Mean ± SD).

Condition	Center Presentation Level (dB SPL)		
	45	55	65
**1**	37.6 ± 19.4	24.3 ± 17.7	30.5 ± 23.4
**2**	80.7 ± 20.1	78.0 ± 23.0	80.1 ± 23.4
**2-1**	44.7 ± 23.2	53.7 ± 21.7	53.0 ± 23.4

**Table 3:** Mean d-values at each presentation level (Mean ± SD).

Condition	Presentation Level (dB SPL)						
	40	45	50	55	60	65	70
**1**	45.3 ± 17.5	39.1 ± 18.1	29.5 ± 13.1	19.3 ± 12.3	27.9 ± 21.3	36.4 ± 32.4	36.1 ± 34.0
**2**	67.4 ± 16.8	65.1 ± 18.7	62.9 ± 19.5	58.1 ± 20.9	63.8 ± 18.6	64.0 ± 18.2	65.3 ± 22.1
**2-1**	27.5 ± 18.0	34.5 ± 16.0	35.9 ± 19.9	40.8 ± 25.3	35.8 ± 23.2	28.6 ± 28.4	29.2 ± 29.0

## Data Availability

The data presented in this study are available upon request from the corresponding author due to privacy concerns.

## Abbreviations

The following abbreviations are used in this manuscript:

UCHL	Unilateral Conductive Hearing Loss
CCHA	Cartilage Conduction Hearing Aids
RMS	Root Mean Square
AC	Air Conduction
IA	Interaural Attenuation
